# Prevalence of Fabry disease in dialysis patients: Western Australia Fabry disease screening study - the FoRWARD study

**DOI:** 10.1186/s13023-019-1290-3

**Published:** 2020-01-13

**Authors:** Sadia Jahan, Subashini Sarathchandran, Shamina Akhter, Jack Goldblatt, Samantha Stark, Douglas Crawford, Andrew Mallett, Mark Thomas

**Affiliations:** 1 Kidney Health Service, Royal Brisbane and Women’s Hospital, Herston, QLD Australia; 2 Faculty of Medicine, The University of Queensland, Brisbane, QLD Australia; 3 Department of Nephrology, Royal Perth Hospital, GPO Box X2213, Perth, WA 6847 Australia; 4 Genetics WA (retired), Subiaco, Australia; 5 National Referral Laboratory (NRL), Adelaide, SA Australia

**Keywords:** Fabry disease, Screening, Dialysis, Dried blood spot, α-GAL-A

## Abstract

**Aim:**

To determine the prevalence of undiagnosed Fabry Disease (FD) in Western Australian (WA) patients undergoing dialysis.

**Background:**

FD is a multisystem X-linked lysosomal storage disease caused by deficient activity of alpha-galactosidase-A (α-GAL-A). Affected individuals are at risk of developing small-fibre neuropathy, rash, progressive kidney disease, hypertrophic cardiomyopathy and ischaemic stroke. Diagnosis is often delayed by years or even decades. Screening high risk population such as dialysis patients may identify patients with undiagnosed Fabry disease.

**Methods:**

A cross-sectional study was undertaken of all adult patients receiving dialysis in WA, without previously known FD. After informed consent they were screened for α-GAL-A activity by dried blood spot samples. Low or inconclusive activity were repeated via Centogene in Rostock, Germany with *GLA* genetic analysis. Ethics approval was granted by Royal Perth Hospital Human Research Ethic Committee REG 14–136; site-specific approval was granted from appropriate authorities; ANZ Clinical Trials Registry U1111–1163-7629.

**Results:**

Between February 2015 & September 2017, α-GAL-A activity was performed on 526 patients at 16 dialysis sites. Twenty-nine patients had initial low α-GAL-A; repeat testing & *GLA* genotyping showed no confirmed FD cases. The causes of false positive rates were thought to be secondary to impaired protein synthesis due to patient malnutrition and chronic inflammation, which is common among dialysis patients, in addition to poor sampling handling.

**Conclusion:**

Analysis of this dialysis population has shown a prevalence of 0% undiagnosed FD. False positives results may occur through impaired protein synthesis and sample handling.

## Background

Fabry disease (FD) is a multisystem X-linked lysosomal storage disease caused by deficient activity of alpha-galactosidase-A (α-GAL-A). This results in an accumulation of glycosphingolipids with terminal α-D-galactosyl residue particularly globotriaosylceramide and globotriaosylsphingosine [1] in organs, namely kidney, heart, peripheral nerves and brain. Symptoms of FD generally emerge during childhood or adolescence with neuropathic pain crisis (acroparesthesia), angiokeratomas, ophthalmologic abnormalities, hypohydrosis and gastrointestinal symptoms. Major organ involvement is from 20 to 30 years of age onwards, leading to chronic kidney disease, hypertrophic/dilated cardiomyopathy, deafness and cerebrovascular diseases.

Affected hemizygous males with no or minimal α-GAL-A activity exhibit a classic phenotype in contrast to heterozygous females, who may display leucocyte α-GAL-A activities within the normal range and can express a range of phenotypes, from asymptomatic to severe symptoms. Its rarity, non-specificity and overlap of α-GAL-A activity with normal range amongst heterozygous women often leads to diagnostic delay or misdiagnosis, negatively impacting a patient’s diagnostic odyssey. Diagnosis in some instances can be delayed by years or decades [2]. Once diagnosed, FD is treatable using Enzyme Replacement Therapy with recombinant human α- galactosidase, which can stabilize or improve affected organ function, decreasing mortality risk from cardiac or cerebral infarction. Affected family members can also be diagnosed through family screening and managed early to prevent irreversible complications.

There have been several global studies estimating the population prevalence of FD [3–5], however these studies have a small sample size, display uniform ethnicity and have variable sample handling and patient selection based often on symptoms leading to suboptimal results. Screening high risk populations such as dialysis patients may especially identify patients with undiagnosed Fabry disease [5]. Due to a significant proportion of end stage kidney disease (ESKD) patients with unknown aetiology having not undergone a renal biopsy; screening studies are essential to determine prevalence, establish early diagnosis and enable potential complication treatment. Therefore, in 2015 we established the FoRWARD study (FabRy testing in Western Australian Renal Dialysis) in order to screen patients receiving dialysis throughout Western Australia. Screening quality was improved by using DBS α-GAL-A assay for better sample handling and diagnosis, involving participants of different ethnicities, and screening all consented dialysis patients regardless of symptoms.

## Methods

All consenting adult patients, without previous diagnosis of FD, undergoing renal replacement therapy throughout Western Australia (WA) were invited to participate. Invitations to recruit participants to the FoRWARD study were sent to all dialysis facilities in Western Australia. The screening was performed by a 3-step method.

In the first step, WA dialysis clinic nurses were educated about the FoRWARD study and consenting process by the lead investigator through an educational video (http://www.researchreview.com/fabrytestingdialysis). Clinical nurses with delegated authority subsequently consented patients using the approved Patient Consent and Information Form (WA Fabry PCIF – V1 5 / simplified WA Fabry PCIF – Kimberley version).

In the second step, routine pre-heparinised dialysis blood samples from consented patients was sent to the PathWest clinical laboratory, where four 3.2 mm blood spots were aliquoted to a Dried Blood Spot (DBS) card. DBS cards were then sent to the National Referral Laboratory (SA Pathology) Adelaide, South Australia, for α-GAL-A enzyme activity testing. α-GAL-A enzyme cut off values in this screening was set with 2.0 nmol/h/ml for both males and females, which has a sensitivity and specificity of 100% for males and a sensitivity of 88% and specificity of 95% for females [6].

In the third step, the test was repeated for patients with low/inconclusive α-GAL-A activity with Lyso- GB3 assay performed for females with equivocal α-GAL-A activity in addition; via Centogene in Rostock, Germany. Those with repeatedly low α-GAL-A enzyme activity and females with high Lyso-GB3 levels underwent Sanger sequencing of the *GLA* gene at Centogene for confirmation of FD.

Test results from all patients were sent to the patient’s supervising physician along with aggregate data to the lead investigator. Patients were informed of their results with confirmed FD cases offered follow-up in the state-wide FD multidisciplinary clinic. Genetic counselling services were also made available for those with positive results to discuss possible familial implications, inheritance pattern and other questions.

Approval was obtained from the Royal Perth Hospital Human Research Ethics Committee REG 14–136. Site-specific approvals were granted from appropriate authorities. This study is registered with ANZ Clinical Trials Registry U1111–1163-7629.

## Results

**Fig. 1:**
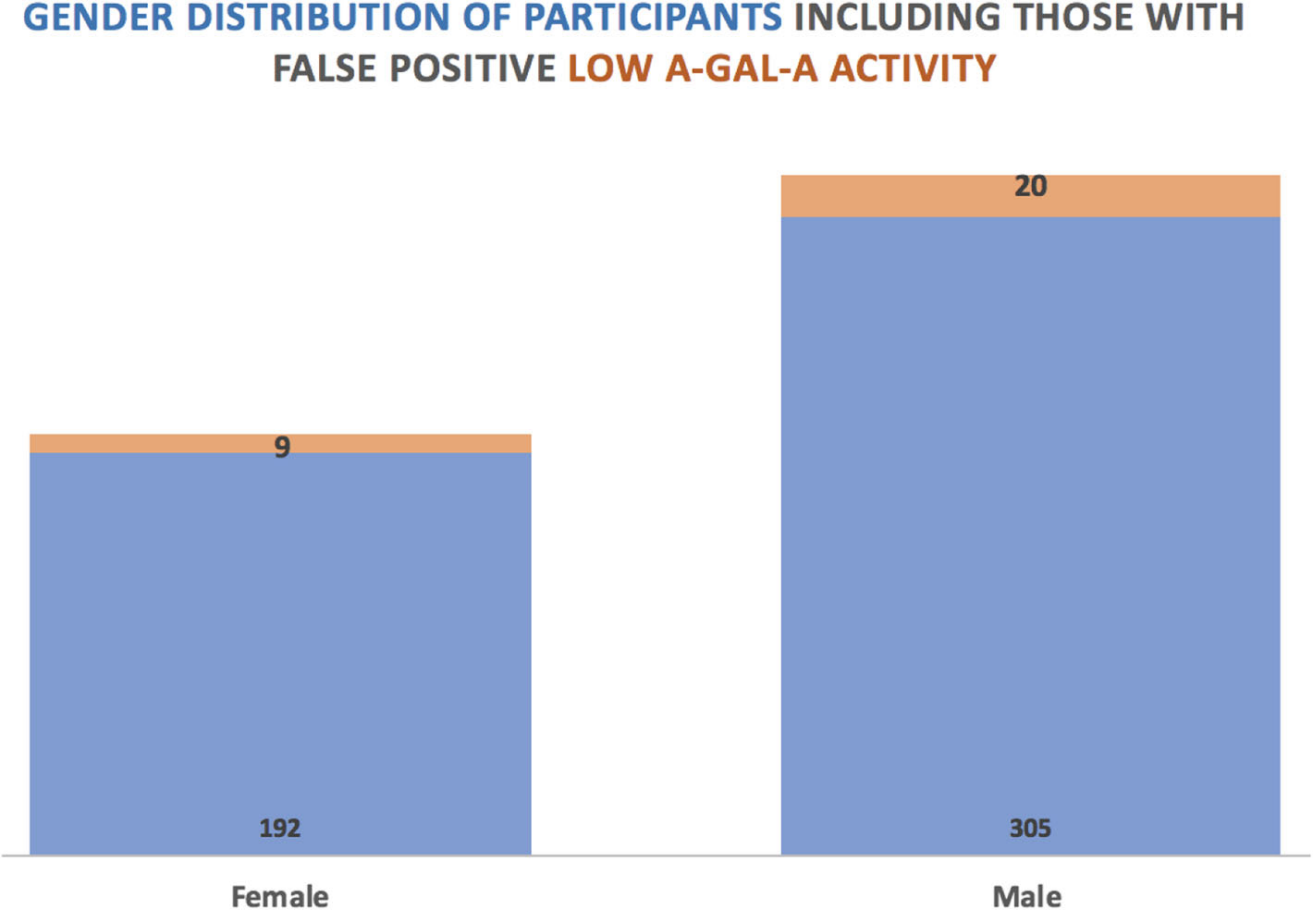
Gender Distribution of Participants including those with false positive low α-GAL-A activity

Between February 2015 to September 2017 (2 years and 7 months) 526 patients were screened in WA. Of these 526 participants across 15 facilities, 325 were male and 201 were female (Fig. 1). Twenty-nine patients from 10 dialysis facilities were found to have α-GAL-A activity < 2 nmol/h/ml, with 20 males and 9 females (Fig. 1). All such patients with low α-GAL-A activity had the test repeated and genetic test performed (Centogene; Germany). Despite low screening α-GAL-A activity detected in NRL DBS assay, all these patients had normal *GLA* genetic studies and normal α-GAL-A activity in Centogene clinical testing.

## Discussion

In this FoRWARD study we did not identify FD in any screened patient, indicating a 0% prevalence of undiagnosed FD amongst the population screened. The false positive rate of the study was 4.5% (9/201) for females and 6.2% (20/325) for males. The causes of these false positive rates of FD screening with DBS assay are unclear. We hypothesis that this may be due to impaired protein synthesis due to patient malnutrition as noted by serum albumin and chronic inflammation, which is common among dialysis patients, in addition to poor sampling handling, especially during postage.

Literature suggests prevalence of FD in haemodialysis patients are higher than general population reporting up to 1.2% [6]. This highlights the importance of considering FD as an aetiology of ESKD.

The FoRWARD study applied highly diagnostic, convenient and inexpensive DBS assay for screening of FD. This method provides a clear delineation between normal female controls and heterozygotes than the leucocyte assay. Due to high sample stability, with weeks as compared to 48 h for the leucocyte assay and simple incubation with low labour cost, more patients were screened. Further, this was able to be undertaken with timing that was more convenient for patients as well as clinical and study staff. Another advantage of the study was screening all consented dialysis patients in WA regardless of their symptoms. This is critical to establish informative results based upon not missing patients with a variety of classical and non-classical phenotypes that could be due to FD as a potential cause of their kidney disease. Also, the multiethnicity of participants further likely reduced study bias given that FD is not limited to certain ethnic groups, although prevalence does exhibit some variation globally.

One limitation of our study was the small-moderate sample size. Previous studies with similar numbers of patients did not identify any patients with FD and therefore we aim to study a bigger population in the future based upon both our findings and those of such other studies. We recommend that other FD screening studies consider anticipated population prevalence in powering sufficiently scaled recruitment in order to reasonably identify a diagnostic signal.

Another limitation of this study includes not deploying genetic testing as the primary screening modality for females. Such female FD heterozygotes can have α-GAL-A activities ranging from affected enzyme activity to the lower limits of the normal range in DBS assay [7]. Given that up to 25% of heterozygotic female participants can have false negative screening results, there is residual chance that female FD cases may have remained undiagnosed. However, the screening quality was significantly improved by repeating the screening test for all females with low or inconclusive α-GAL-A activity and performing Lyso-GB3 assay on females with equivocal α-GAL-A activity to minimise false negative results although noting that these are different results. Such a cascade and tiered screening strategy for females may have utility in addressing the limitations of sole DBS assay testing, particularly when undertaking FD screening at larger scales. Future application of such a screening approach in females might be optimally assessed in a larger recruitment study. We also note the possibility of false negative results with DBS tests as described by previous studies [6].

With the increased accuracy of the FoRWARD study and screening all consented dialysis patients, a higher false positive rate was noted. Therefore, we suggest screening ESKD patients for FD more selectively, based on symptoms, clinical history, or signs rather than merely the diagnosis of ESKD**.** Also, a cost-effective DNA screening of females may be possible with advanced DNA mass spectrometry in populations known to be affected by FD due to restricted numbers of mutations [8]. This may not be feasible in the Australian population due to its multiethnic nature.

## Conclusion

In summary, we did not identify any cases of FD in this dialysis cohort whilst observing a number of false positive screening results through impaired protein synthesis and sample handling. We aim to further study the correlation of α-GAL-A activity with biochemical markers of malnutrition and inflammation, to examine the other suggested causes of false positive α-GAL-A activity.

Additional larger scale studies are required to determine if there is an identifiable prevalence of undiagnosed FD amongst Australian dialysis patients.

## Data Availability

Not applicable.
